# Capitalizing on natural experiments in low- to middle-income countries to explore epigenetic contributions to disease risk in migrant populations

**DOI:** 10.1017/gheg.2015.4

**Published:** 2016-02-05

**Authors:** J. Jaime Miranda, Caren Weinhouse, Rodrigo M. Carrillo-Larco, Lijing L. Yan

**Affiliations:** 1 CRONICAS Centre of Excellence in Chronic Diseases, Universidad Peruana Cayetano Heredia, Lima, Peru; 2 Department of Medicine, School of Medicine, Universidad Peruana Cayetano Heredia, Lima, Peru; 3 Duke Global Health Institute, Duke University, NC, USA; 4 Duke Kunshan University, Kunshan, China

**Keywords:** Chronic disease, epigenetics, global health, migration

Migration poses a significant and worsening public health problem. As the world becomes increasingly interdependent and the global population continues to expand, rates of both within-country and international migration are rising. Migrants tend to experience differential risks for chronic disease, including cardiovascular and metabolic diseases [1–7]. Differential health outcomes in international migrants are not limited to migrants from developing to developed countries; migrants from one developed country to another with regional differences in chronic disease risk may be impacted, as well [8].

Lifestyle factors do not fully explain increased disease risk in some migrant populations. Prior studies have suggested that increases in body mass and blood pressure in migrant populations are related to stress-induced dietary or physical activity changes. These increased risk factors may subsequently influence disease risk [3]. However, individuals that migrated from a subsistence lifestyle on Pacific atoll Tokelau to an urbanized Western lifestyle in New Zealand showed increased blood pressure in men that cannot be fully explained by concomitant dietary changes and weight gain [4]. Migrants often display cardiovascular disease (CVD) risk intermediate to that of non-migrants in their country of origin and to host population natives [5, 9]. These outcomes suggest that setting of origin, together with initial exposures to such settings, plays a role in acquired disease even in the presence of host population lifestyle factors [5, 9]. Although lifetime risks in migrant groups may approach those of the host population over time, there is evidence for differential health outcomes in migrant populations as compared with non-migrants in studies with relatively long follow-up periods. For example, the Finnish Twins Cohort study reported CVD risk intermediate to that of the migrants’ country of origin and of the host population after a 23-year follow-up [5]. Further, in cases in which lifetime risks of migrants do approach the host population over time, the intervening period of differential health is of strong public health interest.

Genetic differences do not fully explain differential disease risk, either. Genetic heterogeneity within a country may contribute to differences in health outcomes between migrants and non-migrants if migration is non-random for genetic markers [8]. However, twins that migrated from Finland to Sweden displayed a higher CVD risk than low-risk native Swedes, but a lower risk than their co-twins in high-risk Finland. These data suggest that differential health by the migration status is strongly influenced by environmental factors [5]. In addition, cardiovascular risk factors in rural-to-urban migrants in Peru were dependent on age at first migration [9]. Individuals that migrated when aged older than 12 years were at higher risk for diabetes and metabolic syndrome as compared with individuals that migrated at younger ages [9]. Individuals in both groups are likely genetically similar on the population level and have experienced similar environments, albeit at different life course stages.

Therefore, we propose that epigenetic reprogramming due to early life environment may contribute to differential chronic disease risk in migrant populations. This effect may be strongest in rural-to-urban migrants that experience significantly different environments across the life course. Current evidence supports epigenetic mediation of the link between developmental exposures and metabolic dysfunction and CVD [10], health outcomes with common differential risk in migrant populations as compared with non-migrants.

Epigenetic modifications can cause a change in phenotype with no change in underlying genotype. The epigenome, or genome-wide collection of epigenetic modifications, consists of somatically heritable gene regulatory marks, including DNA methylation, posttranslational histone tail modifications, and chromatin remodeling proteins [11]. The field of environmental epigenetics, or the study of epigenetic responsiveness to the external environment, may partly explain the developmental origins of health and disease, or inter-individual variation in health outcomes in adulthood based on environmental exposures during early life development. [11].

Low- and middle-income countries in the developing world are ideal locations for studying epigenetic contributions to migrant health due to rising urbanization and emigration. Based on our research expertise, we propose Latin America and China as study sites for epigenetic questions in migrant populations. Latin American urbanization rates have risen very quickly in recent decades. In fact, the rural population is now two-thirds smaller than it was in the 1950s [12, 13]. China has experienced similar massive internal migration over the last 30 years, with millions of people relocating from rural to urban areas, offering potentially large study populations [14]. In addition, both Latin America and China are large and topographically diverse regions, allowing for setting-specific studies of rural-to-urban migrants coming from different locations, such as the coast or highlands, and from different altitudes [9, 14]. Lastly, migrants coming to urban settings for socioeconomic purposes are not identical to those who have migrated to flee earthquakes and floods or violence [9]. Latin American and China have both experienced historical political unrest and natural disasters that invite studies of multiple motivations for migration [9, 14].

Most environmental epigenetic research has focused on social, nutritional, and chemical exposures, rather than demographic shifts. To the best of our knowledge, only one published study explores epigenetic profiles of migrants to date, specifically, within-country migrants in Italy [15]. None have been published in the developing world. Therefore, systematic studies of international and within-country migration represent a timely and important opportunity to investigate a potential role for the epigenome in altered chronic disease risk of migrants.
